# Transcriptome Analysis Reveals the Mechanism of Y0-C10-HSL on Biofilm Formation and Motility of *Pseudomonas aeruginosa*

**DOI:** 10.3390/ph17121719

**Published:** 2024-12-19

**Authors:** Deping Tang, Yali Liu, Huihui Yao, Yanyan Lin, Yanpeng Xi, Mengjiao Li, Aihong Mao

**Affiliations:** 1 School of Biological & Pharmaceutical Engineering, Lanzhou Jiaotong University, Lanzhou 730070, China; tangdp@mail.lzjtu.cn (D.T.);; 2 Gansu Provincial Academic Institute for Medical Research, Lanzhou 730050, China

**Keywords:** *P. aeruginosa*, biofilm, transcriptome, quorum sensing inhibitor, *C. elegans*

## Abstract

**Background:** *Pseudomonas aeruginosa* (*P. aeruginosa*) is a type of pathogen that takes advantage of opportunities to infect and form biofilm during infection. Inhibiting biofilm formation is a promising approach for the treatment of biofilm-related infections. **Methods:** Here, Y0-C10-HSL (N-cyclopentyl-n-decanamide) was designed, synthesized, and tested for its effect on biofilm formation, motility, and the *Caenorhabditis elegans* (*C. elegans*) survival assay. In addition, the molecular mechanism of Y0-C10-HSL on *P. aeruginosa* biofilm formation was explored using transcriptome analysis. **Results:** At a concentration of 200 μmol/L Y0-C10-HSL, biofilm and exopolysaccharides were decreased by 38.5% and 29.3%, respectively; Y0-C10-HSL effectively dispersed the pre-formed biofilm and inhibited the motility ability of *P. aeruginosa*; and the *C. elegans* survival assay showed that Y0-C10-HSL was safe and provided protection to *C. elegans* against *P. aeruginosa* infection (the survival rates of *C. elegans* were higher than 74% and increased by 39%, 35.1%, and 47.5%, respectively, when treated with 200 μmol/L Y0-C10-HSL at 24, 48, and 80 h). Transcriptome analysis showed that 585 differentially expressed genes (DEGs) were found after treatment with 200 μmol/L Y0-C10-HSL, including 254 up-regulated DEGs and 331 down-regulated DEGs. The genes involved in the quorum sensing system and biofilm formation were down-regulated. **Conclusions:** Y0-C10-HSL inhibited the biofilm formation and dispersed the pre-formed biofilm of *P. aeruginosa* through down-regulated genes related to quorum sensing pathways and biofilm formation. These findings provide a theoretical foundation for the treatment and prevention of antibiotic resistance in clinical and environmental microorganisms such as *P. aeruginosa*.

## 1. Introduction

The survival selection pressure caused by antibiotic-mediated bactericidal and bacteriostatic activity is one of the important factors that induce bacteria to develop drug resistance. Antimicrobial resistance (AMR) has been recognized as a significant global clinical issue. With the discovery of antibiotics, infection is no longer as fatal a problem for clinical doctors as it has been in the past. However, the distribution of a large number of mobile genetic elements and related resistance genes, as well as the overuse of antibiotics, have led to the rapid spread of AMR worldwide [1]. Therefore, the quorum sensing (QS) system of bacteria has gained the attention of an increasing number of researchers. QS is a bacterial density-dependent intercellular communication mechanism that can activate signaling pathways in bacteria, host cells, and regulate a variety of biological properties, such as bacterial motility, virulence, and biofilm formation [2,3,4]. *Pseudomonas aeruginosa* (*P. aeruginosa*) is a Gram-negative facultative anaerobic bacterium that can grow in a diverse range of environments. It is also a major opportunistic pathogen involved in diverse acute and chronic diseases, such as cystic fibrosis (CF), human immunodeficiency virus pneumonia, urinary tract infections, otitis externa, bacteremia, and burns [5,6]. Importantly, *P. aeruginosa* causes severe infections in patients with immune insufficiency (such as those with severe burns, diabetes, cancer, and organ transplantation) and long-term intubation of chronic diseases, resulting in high infection morbidity and mortality. The QS system of *P. aeruginosa* regulates approximately 4% to 6% of gene expression, including over 30 virulence factors related to its pathogenicity (such as proteases, elastases, iron carriers, exotoxins, and rhamnolipids), as well as biofilm formation and diffusion associated with multidrug resistance and chronic infection [5,6,7]. The QS system of *P. aeruginosa* consists of four closely related subsystems, namely Las, Rhl, Pseudomonas quinolone signaling (PQS), and the integrated quorum sensing system (IQS), which produce homologous autoinducer (AI) molecules within the cell, namely N-3-oxo-dodecanoyl-L-hydroserine lactone (3-O-C_12_-HSL), N-butyryl-L-hydroserine lactone (C_4_-HSL), 2-heptanyl-3-hydroxy-4-quinolone (PQS), and 2-(2-hydroxyphenyl)-thiazol-4-aldehyde (IQS) [8]. The chemical structures of the signal molecules of each subsystem are shown in Table 1. The Las system and Rhl system belong to N-acyl-homoserine lactone (AHL)-mediated systems, which are composed of LuxI synthase and LuxR receptor proteins. In the Las system, LasI synthesizes 3-O-C_12_-HSL as the AI of the system. When it reaches a certain threshold, the molecule binds to the LasR receptor protein, thereby activating it and forming the LasR-3-O-C_12_-HSL complex, which directly or indirectly regulates gene expression. The distinction between the Rhl system and the Las system is that the AI in the Rhl system is C_4_-HSL, and the complex is RhlR-C_4_-HSL. In *P. aeruginosa*, the PQS system is exclusive and employs 2-alkyl-4-quinolones (AQs) as the signaling molecule. These AQs include 2-heptyl-3-hydroxy-4-quinolone (PQS) and its precursor 2-heptyl-4-hydroxyquinoline (HHQ) [9,10]. The IQS system, whose signaling molecules are called IQS, has the function of integrating QS signals and stress responses. There are mutual regulatory effects among various subsystems, which are interrelated and form a multi-level and intricate network regulatory circuit, ultimately leading to bacterial infection in the host organism. In summary, the inhibition of the QS system and pathogenicity represents a highly promising avenue for the investigation of anti-virulence compounds.

Multidrug-resistant (MDR) and extensively drug-resistant (XDR) *P. aeruginosa* isolates are a common source of serious nosocomial infections and a significant contributor to morbidity and mortality. According to statistics from the Centers for Disease Control and Prevention, 8.9% of *P. aeruginosa* isolates in the year 2021 appear to be MDR [11]. Additionally, the abundance of virulence factors and sophisticated antibiotic resistance pathways make *P. aeruginosa* a highly clinically significant pathogen that requires effective management [12]. Consequently, it has become a crucial pathogen in the development of novel antibacterial agents in recent years. At present, the primary therapeutic strategy for *P. aeruginosa* infection involves the administration of various antibiotics, including tazobactam, tobramycin, gentamicin, ciprofloxacin, levofloxacin, imipenem, and meropenem [13,14,15,16]. However, the traditional antibiotic therapy based on bactericidal or antibacterial strategies has led to a variety of resistant bacteria, including MDR or even pan-resistant *P. aeruginosa*, posing a huge challenge to clinical treatment. Therefore, the search for new antibacterial drugs and treatment methods is of the utmost importance. Quorum sensing inhibitors (QSIs) can weaken bacterial virulence and boost the susceptibility of bacterial biofilm to antibiotic therapy without affecting bacterial growth. These characteristics have made QSIs a prominent area of research and development within the field of infection control. There have been reports on the artificial synthesis of *P. aeruginosa* QSIs, such as halogenated furanone derivatives [17], novel quinazolinone disulfide analogues [18], phenylpropanoid heterocyclic derivatives [19], and novel L-glucosamine lactone [20]. In the study, an AHLs analogue (N-cyclopentyl-n-decanamide, Y0-C10-HSL) was designed and synthesized. The QSI effect and antibacterial mechanism were investigated in the hope of providing a theoretical basis to help in the treatment of *P. aeruginosa* infection, with a view to finding new possible drugs for treating infection.

## 2. Results

### 2.1. Synthesis of Y0-C10-HSL

**Figure 1:**
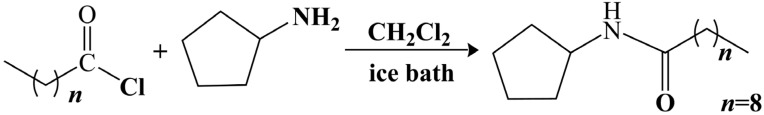
Chemical synthesis of compound Y0-C10-HSL. Cyclopentylamine and decanoyl chloride were reacted at 0 °C for 30 min and room temperature for 14 h. Y0-C10-HSL (N-cyclopentyl-n-decanamide) was isolated using the extraction method.

AHLs are the most commonly utilized molecules as quorum sensing autoinducers in Gram-negative bacteria. They are comprised of an invariant homoserine lactone ring and an acyl chain. AHLs can vary not only in the length of the acyl chain but also in relation to the saturation state of their acyl chain and the oxidation state of position 3 [21]. We synthesized Y0-C10-HSL by reconstructing the homoserine lactone ring and the acyl chain of the natural signaling molecules (3-O-C_12_-HSL and C_4_-HSL). Figure 1 illustrates the chemical synthesis of Y0-C10-HSL. Since Y0-C10-HSL is insoluble in water, we first dissolved it in DMSO to achieve a concentration of 100 mmol/L. For subsequent use, we added the mother solution to the culture medium and diluted it to the desired concentration. It has been shown that DMSO, an excellent co-solvent, does not interfere with bacterial growth at concentrations below 1% [22,23]. To minimize the impact of DMSO throughout the experiment, we maintained the concentration of DMSO in the culture system at 0.2%.

### 2.2. Growth Curves

**Figure 2:**
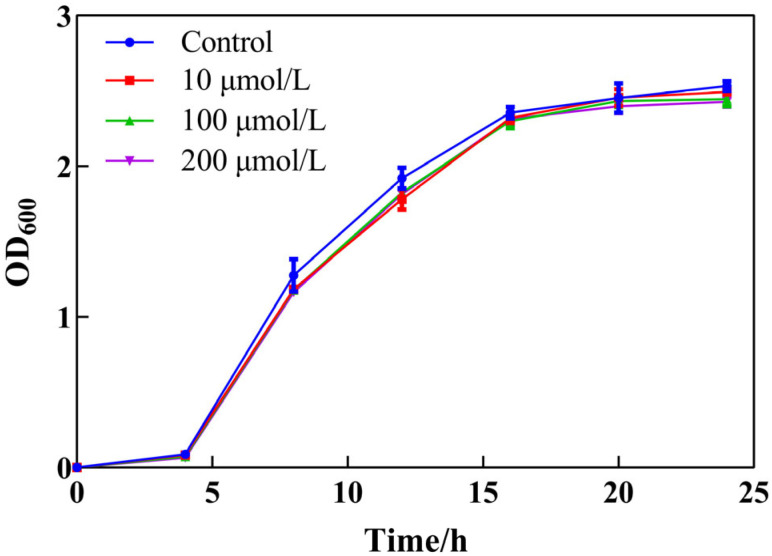
Effect of different concentrations of Y0-C10-HSL on the growth curve of *P. aeruginosa*. Bacteria were cultured in a PPGAS medium at 37 °C and 150 rpm for 24 h under 0, 10, 100, and 200 μmol/L Y0-C10-HSL, respectively. OD_600_ was measured every 2 h.

In order to study the regulatory capacity of Y0-C10-HSL on the biofilm, growth curves of *P. aeruginosa* under Y0-C10-HSL were conducted. Figure 2 shows that Y0-C10-HSL had no inhibition effect on the growth of *P. aeruginosa* ranging from 10 to 200 μmol/L.

### 2.3. The Effect of Y0-C10-HSL on Biofilm

**Figure 3:**
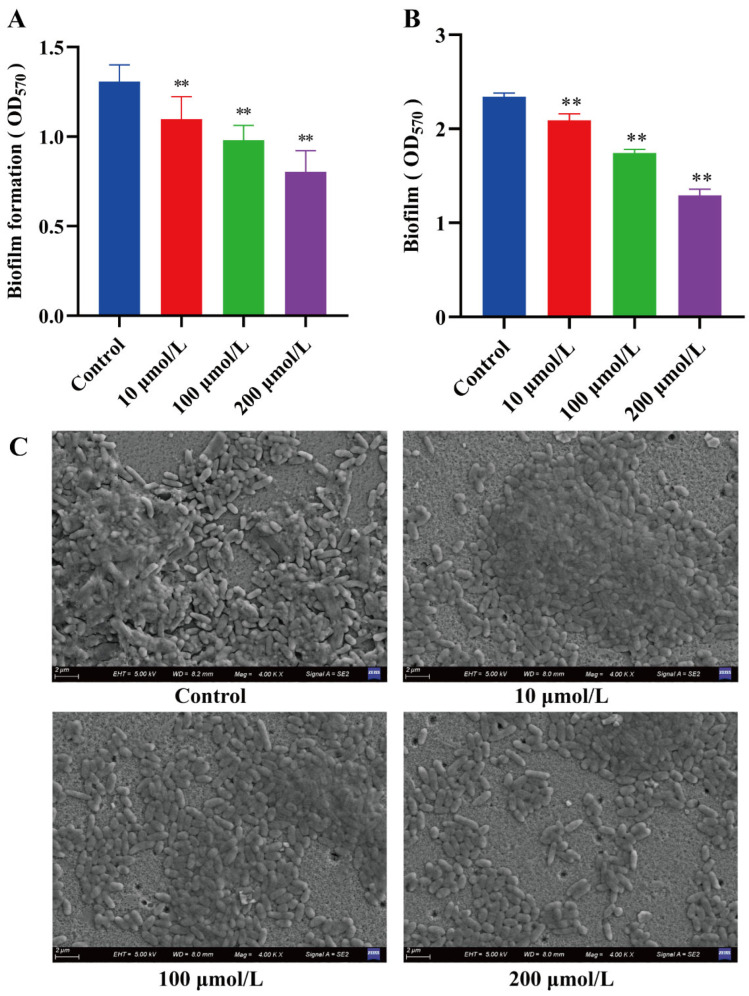
Effect of Y0-C10-HSL on *P. aeruginosa* biofilm. (**A**) Effect of Y0-C10-HSL on biofilm formation of *P. aeruginosa*. (**B**) The dispersion effect of Y0-C10-HSL on pre-formed biofilm of *P. aeruginosa*. (**C**) Effect of Y0-C10-HSL on the structure of biofilm. The analysis method used was a *t*-test; **: significant difference (*p* < 0.01).

*P. aeruginosa,* an opportunistic human pathogen, is well known for its ability to persist in hospital settings due to its capacity to develop resilient biofilm that is resistant to antibiotics [8]. The development of biofilm by *P. aeruginosa* hinders the treatment by providing protection to bacterial cells against environmental stress, antibiotics, and phagocytosis, thereby enabling colonization and long-term persistence [8,24]. In the study, we evaluated the potential role of Y0-C10-HSL in both *P. aeruginosa* biofilm development and dispersion of pre-formed biofilm. As shown in Figure 3A, Y0-C10-HSL significantly inhibited the biofilm formation of *P. aeruginosa* in a dose-dependent manner. The biofilm formation was reduced by approximately 15.9%, 25%, and 38.5% at the concentrations of 10, 100, and 200 μmol/L Y0-C10-HSL, respectively (*p* < 0.01). In order to investigate whether the compound has a dispersing effect on pre-biofilm, the dispersion effect of Y0-C10-HSL on the pre-formed biofilm of *P. aeruginosa* was conducted. Figure 3B demonstrates that the dispersibility of pre-formed biofilm also showed a dose-dependent manner. The biofilm was decreased nearly 10.7%, 25.6%, and 44.8% at the concentrations of 10, 100, and 200 μmol/L Y0-C10-HSL, respectively (*p* < 0.01). Finally, the impact of Y0-C10-HSL on the structure of biofilm was visualized using scanning electron microscopy (SEM), and the images are presented in Figure 3C. It was observed that the control group exhibited a dense polysaccharide matrix, with a large number of bacterial cells overlapping and gathering to form a thick biofilm. In contrast, at the concentration of 200 μmol/L Y0-C10-HSL, the biofilm appeared noticeably sparse, with cells being extensively liberated and the clumps of cells being significantly smaller compared to the control group. In conclusion, these results demonstrate that Y0-C10-HSL inhibits biofilm formation and facilitates the dispersal of pre-formed biofilm of *P. aeruginosa*.

### 2.4. The Effect of Y0-C10-HSL on Exopolysaccharides

**Figure 4:**
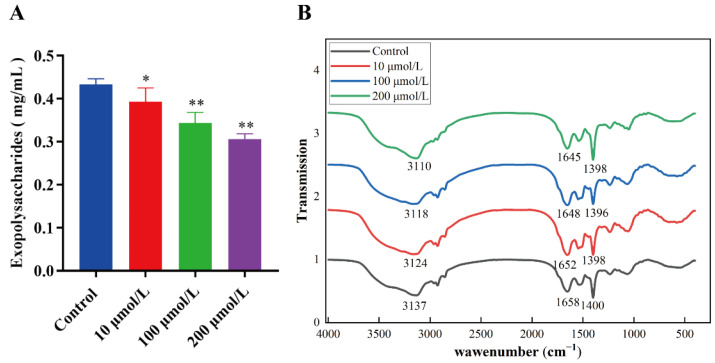
Effect of Y0-C10-HSL on the surface chemical groups of extracellular polymers and exopolysaccharides of *P. aeruginosa*. (**A**) Effect of Y0-C10-HSL on exopolysaccharides. (**B**) Effect of Y0-C10-HSL on the surface chemical groups of extracellular polymers. The analysis method used was a *t*-test; *: significant difference (0.01 < *p* < 0.05); **: significant difference (*p* < 0.01).

Exopolysaccharides are the primary component of bacterial biofilm and play a pivotal role in its development. As a member of the biofilm matrix, *P. aeruginosa* is capable of secreting three types of exopolysaccharides, namely alginate, Pel, and Psl, each serving a unique function in facilitating biofilm development and evading immune responses during infection [25,26]. The effect of Y0-C10-HSL on the exopolysaccharides of *P. aeruginosa* is shown in Figure 4A. In comparison to the control group, the production of exopolysaccharides was significantly inhibited by Y0-C10-HSL (*p* < 0.05). The inhibition rate of exopolysaccharides production was 9.63%, 20.76%, and 29.3% at 10, 100, and 200 μmol/L, respectively. FT-IR is a widely employed technique for the analysis of functional groups. Previous studies have demonstrated a strong correlation between the peak intensity in the infrared spectrum and the concentration of functional groups within samples, thereby enabling an accurate reflection of their relative concentrations. As illustrated in Figure 4B, many absorption peaks were identified within the 500~4000 cm^−1^ range. The corresponding infrared absorption peaks in the control group and the treatment groups with different concentrations were observed to be in similar positions, indicating that the major functional group classes were basically identical. The peaks observed at the 3400 cm^−1^ beams of the spectrum are typically attributed to the stretching vibration of O-H and N-H groups. The hydroxyl compounds can associate, which causes the absorption peak of the O-H group to shift towards the lower wave number. It has been reported that the absorption peaks at 1600~1700 cm^−1^, 1500~1600 cm^−1^, and 1200~1300 cm^−1^ are produced by the amide I band, amide II band, and amide III band, respectively, which are closely related to the protein group [27,28]. The absorption peak at 1400 cm^−1^ is attributed to the stretching vibration of C=O, which is linked to the bacterial cell wall peptidoglycan and capsule. When treated with different concentrations of Y0-C10-HSL, the vibrational characteristics at each wave number exhibited changes. A broad and intense absorption peak emerged in the range of 3400 to 3100 cm^−1^, attributed to O-H stretching vibrations, indicating the presence of polysaccharides within EPS. In comparison to the control group, significant alterations were observed in the vibrational characteristics for the treatment groups at concentrations of 10, 100, and 200 μmol/L. Notably, the characteristic absorption peak of polysaccharides shifted from 3137 cm^−1^ to 3124 cm^−1^, 3118 cm^−1^, and 3110 cm^−1^, respectively. The peaks associated with the amide I band resulting from C=C and C=O stretching vibrations also demonstrated a shift from 1658 cm^−1^ to values of 1652 cm^−1^, 1648 cm^−1^, and 1645 cm^−1^. Furthermore, a notable alteration was observed at around 1400 cm^−1^, where the intensity of the peak gradually decreased with an increase in Y0-C10-HSL concentration. To summarize, Y0-C10-HSL mainly impacts the O-H, N-H, C=C, and C=O functional groups on the EPS surface.

### 2.5. The Effect of Y0-C10-HSL on the Motility

**Figure 5:**
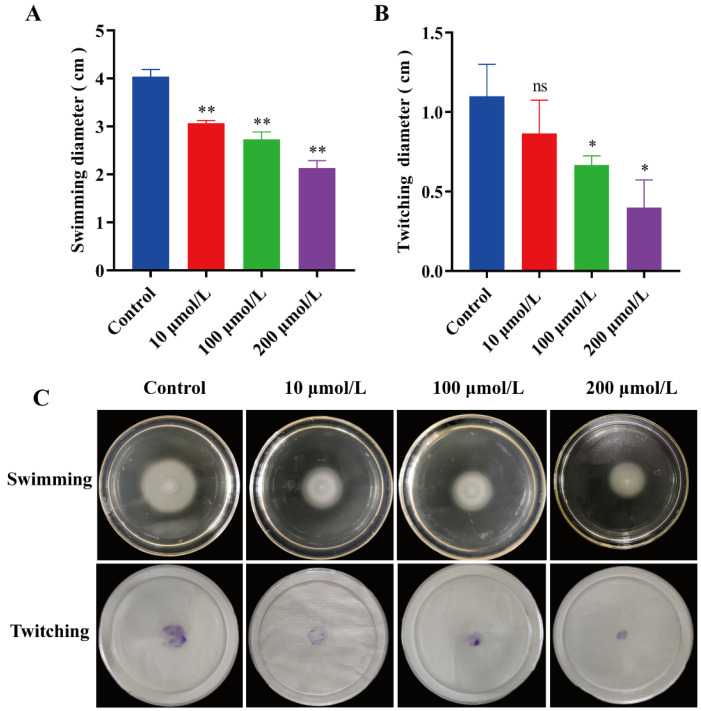
Effect of Y0-C10-HSL on the motility of *P. aeruginosa*. (**A**) Swimming distance of each group. (**B**) Twitching diameter of each group. (**C**) Swimming motility and twitching motility. The analysis method used was a *t*-test; ns: no significant difference (*p* > 0.05); *: significant difference (0.01 < *p* < 0.05); **: significant difference *(p* < 0.01).

Motility plays a significant role in the initial attachment of bacterial cells to both biological and abiotic surfaces, thereby facilitating biofilm formation. Swimming and twitching motilities are necessary factors for biofilm formation; they allow for bacterial chemotaxis, connection, and adhesion to surfaces, thus enabling biofilm formation [29,30]. We further studied the impact of Y0-C10-HSL on *P. aeruginosa* motility. Figure 5 shows that Y0-C10-HSL exhibited varying degrees of inhibition on swimming and twitching motilities. The effect of swimming motility of *P. aeruginosa* is shown in Figure 5A,C, where the results of swimming motility of *P. aeruginosa* showed foggy concentric circles centered on the inoculation point on the agar plate. Y0-C10-HSL exhibited a significant dose-dependent inhibitory effect on swimming motility. The inhibition rate was 24%, 32.2%, and 47.1% at the concentrations of 10, 100, and 200 μmol/L Y0-C10-HSL, respectively (*p* < 0.01). The result of twitching motility is shown in Figure 5B,C, where the inoculum points in the center formed a disc-like structure. The inhibition effect was relatively weak, and there appeared to be no significant difference at the concentration of 10 μmol/L (*p* > 0.05), while there was significant inhibition at concentrations of 100 μmol/L and 200 μmol/L Y0-C10-HSL.

### 2.6. The Effect of Y0-C10-HSL on C. elegans Survival

**Figure 6:**
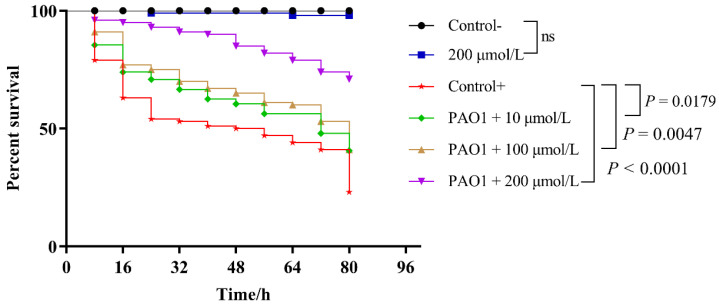
Effect of Y0-C10-HSL on *C. elegans* survival assay. Nematode Growth Medium (NGM) plate contained different concentrations of Y0-C10-HSL (10, 100, and 200 μmol/L) was inoculated with *E. coli* OP50 and *P. aeruginosa*; after culturing for 24 h, L4-stage *C. elegans* worms were transferred onto the plate. *C. elegans* survival assay was measured every 8 h.

*Caenorhabditis elegans* (*C. elegans*) is a potent model animal for studying developmental biology and host–pathogen interaction; it is usually used as a host because of its in-depth characterization and experimental simplicity [31]. More importantly, *C. elegans* serves as a wonderful model organism for research in biology and drug screening due to the conservation of numerous genes and innate immune pathways between *C. elegans* and humans [32]. Therefore, it is imperative to explore the impact of Y0-C10-HSL on *C. elegans* as the chosen model organism in order to enhance the scientific rigor and academic relevance of this study. In this study, we first prepared NGM plates containing different concentrations of Y0-C10-HSL. Then, they were inoculated with *E. coli* OP50 or *P. aeruginosa* and incubated for 24 h. Finally, nematodes were inoculated onto them to establish different groups of *C. elegans* models. The effect of Y0-C10-HSL on the survival state of *C. elegans* infected with *P. aeruginosa* is shown in Figure 6. Excitingly, a concentration of 200 μmol/L of Y0-C10-HSL demonstrated no impact on the survival of *C. elegans*. Within 80 h, the nematodes in the negative control group were in good growth condition and the mortality rate was 0%, while for the nematodes in the positive control group, which was not given Y0-C10-HSL treatment, the mortality rate was 45.7% at 24 h. With the prolongation of the time, there was a greater number of nematode deaths, and by 80 h, the nematode survival rate was only 23.5%. When different concentrations of compounds were given, the nematode mortality rate gradually decreased compared to the positive control. At the three different time points of 24 h, 48 h, and 72 h, the survival rates of nematodes in the 10 μmol/L treatment group were 71.8%, 61.7%, and 45.9%, respectively; the survival rates of nematodes in the 100 μmol/L treatment group were 74.7%, 65.4%, and 53.0%, respectively; and the survival rates of nematodes in the 200 μmol/L treatment group were 93.3%, 84.8%, and 74.0%, respectively. Therefore, it can be seen that the protective effect of Y0-C10-HSL on nematodes exhibits a dose-dependent manner.

### 2.7. Transcriptome Data Quality Analysis

RNA concentration, integrity, and RNA integrity number (RIN) values are shown in Table S1. The RIN values were ≥9.4 (≥8.0), OD260/280 was ≥2.04 (≥1.8), and OD260/230 was ≥2.06 (≥1.0). Table S1 shows that the RNA quality and quantity conformed to the normal requirements for library preparation and transcriptome analysis. The quality of the sequencing is presented in Table S2. Q20 and Q30 are key indicators for assessing base quality. When Q20 > 90% and Q30 > 85%, the sequencing quality is deemed satisfactory. Table S2 shows that the control group had a raw Q20 greater than 95.67% and a raw Q30 greater than 91.5%. The treatment group had a raw Q20 greater than 95.56% and a raw Q30 greater than 92.8%. Both groups had clean Q20 and clean Q30 values exceeding 94%. Overall, the results indicated that the sequencing quality was satisfactory, and the obtained sequences were highly reliable. In addition, the comparison rate with the reference genome is a crucial metric for assessing the data quality of the project data. The clean reads from quality control were aligned to the reference genome using Bowtie2, and gene information was compared as presented in Table S3. The comparison rate with the reference gene exceeded 80%, indicating a high level of concordance. Moreover, the average number of uniq mapped reads was 29,845,972 (82.32%) for the control group and 27,696,953 (81.10%) for the experimental group; when the alignment rate of uniq mapped reads is over 70%, it is usually accepted as ready for follow-up analysis. Table S3 shows that the alignment results satisfied the experimental demands. The correlation of gene expression levels between samples reflects the similarity of expression levels among different treated samples, which is often employed to assess experimental reliability and sample selection rationality. A higher correlation coefficient value approaching 1 indicates greater similarity between samples and fewer differential genes among them. To depict the gene expression correlation between samples, a heat map was obtained using the Pearson correlation coefficient to evaluate the relationship between 0.3% DMSO (Y1_1, Y1_2, Y1_3) and treatment groups (Y2_1, Y2_2, Y2_3). As shown in Figure S3, it can be seen that the Pearson coefficient in the group was higher than 0.972, and R2 was greater than 0.8, which proves that the samples had a strong correlation and good repeatability.

### 2.8. Transcriptome Analysis

**Figure 7:**
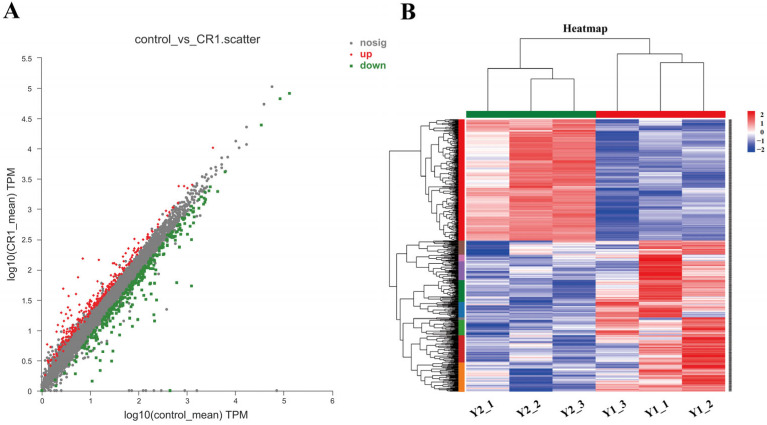
Differentially expressed gene map. (**A**) Scatter plot of differentially expressed genes. (**B**) Heat map of differentially expressed genes. Red indicates up-regulated genes, blue indicates down-regulated genes.

We employed the DESeq2 method for detecting differentially expressed genes (DEGs) across the test groups. The screening thresholds for DEGs were set as follows: *p*-value < 0.05 and FC (fold change) > 1.5. In total, 549 significant DEGs were screened, among them 245 up-regulated genes and 304 down-regulated genes (Table S4). Both scatter plot analysis and heat map visualization demonstrated a substantial impact of compound Y0-C10-HSL treatment on gene expression in *P. aeruginosa* PAO1 (Figure 7).

GO is a tool provided to elucidate the relationship between genes and gene products and is a standardized classification system for gene function. GO annotation facilitates our comprehension of the biological significance conveyed by genes, encompassing three ontologies that describe biological processes, molecular functions, and cellular components. In the study, we conducted GO enrichment analysis on 245 up-regulated genes and 301 down-regulated genes in *P. aeruginosa*. Figure 7A,B show the top 20 GO functional descriptions with high levels of enrichment of down-regulated and up-regulated DEGs, respectively. Among them, the most abundant down-regulated mRNA enrichment included ATP-dependent activity (21), organic acid transport (13), amino acid transport (12), and ABC-type transporter activity (9). The most abundant down-regulated mRNA enrichment included response to stimulus (23), cellular response to stimulus (17), cellular response to stress (13), and homeostatic process (8).

**Figure 8:**
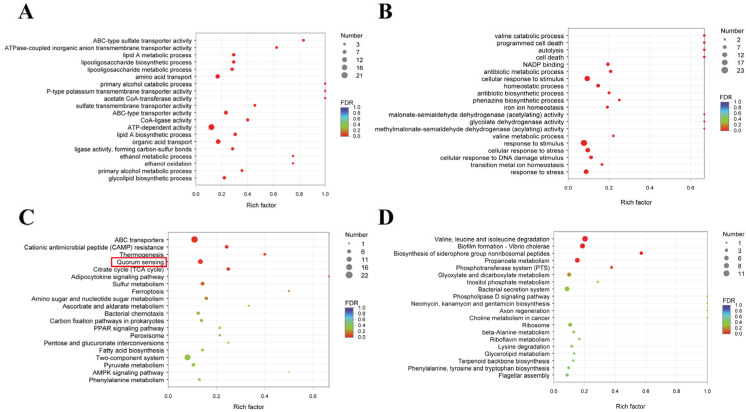
Enrichment analysis of the differentially expressed genes. (**A**) GO enrichment analysis of down-regulated DEGs. (**B**) GO enrichment analysis of up-regulated DEGs. (**C**) KEGG enrichment analysis of down-regulated DEGs. (**D**) KEGG enrichment analysis of up-regulated DEGs.

The KEGG database serves as a comprehensive knowledge repository for the systematic analysis of gene functions and the integration of genomic and functional information. Similar to GO enrichment analysis, we conducted KEGG enrichment analysis on 254 up-regulated genes and 331 down-regulated genes in *P. aeruginosa* (Figure 8). Figure 8C,D showed the top 20 pathways with higher KEGG enrichment for down-regulated and up-regulated DEGs, respectively. The top four pathways in which down-regulated DEGs were significantly enriched included ABC transporters (22), the two-component system (19), quorum sensing (13), and sulfur metabolism (8). The top four pathways in which up-regulated DEGs were significantly enriched included valine, leucine, and isoleucine degradation (11), propanoate metabolism (8), the bacterial secretion system (8), and glyoxylate and dicarboxylate metabolism (6). Excitingly, 13 mRNAs were enriched for pathways in the QS system in the KEGG enrichment analysis. The DEGs down-regulated in association with the *P. aeruginosa* QS system and biofilm are shown in Table 2. The genes involved in the movement were fleP, *BGV84_RS01600*, and *BGV84_RS23030*. The genes associated with biofilm formation were *BGV84_RS17725*, *BGV84 _ RS13310*, *tssC*, and *crp*. QS-related genes were *GV84_RS03095*, *BGV84_RS03090*, *BGV84_RS25465*, *BGV84_RS19945*, *pmrB*, *livG*, *BGV84_RS10805*, *phzA*, *fadD1*, *BGV84_RS03100*, *crp*, *potA*, and *BGV84_RS17185*. It was noted that the *phzA* gene was involved in the synthesis of pyocyanin.

Many important bacterial virulence factors are secreted and closely connected to the secretion system. The type VI secretion system (T6SS) was found to be essential in the pathogenic mechanism associated with Gram-negative bacteria [33]. *P. aeruginosa* has the capability to encode three T6SS clusters, designated H1-, H2-, and H3-T6SS. Hcp (hemolysin coregulated protein) is an important pipeline structural protein in T6SS. It belongs to the same super-protein family as VgrG and is the structural basis for the function of T6SS [34]. BGV84_RS17725, BGV84_RS13310, and tssC were down-regulated by Y0-C10-HSL. It is noteworthy that they all function as Hcp family type VI secretion effectors. Hcp also plays important roles as an effector protein in bacterial adhesion and invasion, phagocyte survival, host immune response, and pathogenicity [34,35,36]. The study by Chen et al. reveals that T6SS plays a contributory role in the formation of *P. aeruginosa* biofilm [37]. Conclusively, it has been demonstrated that Y0-C10-HSL can down-regulate the expression of genes corresponding to T6SS effectors, which is disadvantageous to the development of *P. aeruginosa* biofilm.

## 3. Discussion

It is interesting that *P. aeruginosa* has demonstrated high inherent resistance to most antibiotics, and the selective pressure imposed by antibiotic-induced bactericidal and bacteriostatic activities serves as a significant driving force for the development of bacterial resistance [2,38]. The synthesis of Y0-C10-HSL involves simultaneous modification of the homoserine lactone ring and acyl side chain of the AHL signal molecule. Upon administration, this drug exhibits a remarkable inhibitory effect on biofilm formation, exopolysaccharide secretion, as well as swimming and twitching motilities in *P. aeruginosa*. It is intriguing that Y0-C10-HSL does not exert any discernible impact on the normal growth of *P. aeruginosa* cells. Takenori Ishida and colleagues also demonstrated that the growth of *P. aeruginosa* was not affected at the compound concentration of 250 μmol/L [39]. Therefore, we can infer that Y0-C10-HSL has the advantage of being less likely to cause pressure on bacterial survival as well as to trigger bacterial resistance, making it a promising antimicrobial agent.

The drug resistance mechanism of *P. aeruginosa* can be classified into three types: (ⅰ) endogenous drug resistance, which includes reducing the permeability of the outer membrane, pumping out antibiotics by efflux pump, and producing inactivated enzymes; (ⅱ) acquired drug resistance, which mainly includes self-mutation and obtaining foreign drug resistance genes; and (ⅲ) adaptive drug resistance, which mainly includes the development of bacterial biofilm and the existence of persistent bacteria [40]. The adaptive resistance of bacteria, resulting from transient changes in gene and/or protein expression in reaction to the stimulus of the environment, enhances their ability to withstand antibiotic attack, and this phenomenon is reversible upon removal of the stimuli [40,41]. In the case of *P. aeruginosa*, the most prominent mechanism of adaptive resistance lies in biofilm formation and the emergence of persistent cells, thereby resulting in prolonged infection and an unfavorable prognosis among patients with cystic fibrosis. Consequently, utilizing biofilm as a focal point is the key to treat and prevent *P. aeruginosa* infection. We found that Y0-C10-HSL could inhibit biofilm formation and disperse the pre-formed biofilm in a dose-dependent manner. Extracellular DNA (eDNA) is a crucial constituent of the biofilm matrix, and its degradation has been demonstrated to significantly weaken this matrix. Previous studies have indicated that EndA is particularly important in eDNA degradation, which is essential for dispersing existing biofilm [42]. Therefore, we hypothesize that the dispersal effect of Y0-C10-HSL on biofilm may be attributed to its potential promotion of eDNA degradation. Pel polysaccharides are also able to cooperate with eDNA through cation–anion interactions within the biofilm matrix, thereby solidifying the structure of the biofilm [8]. If more eDNA is degraded as well as the inhibitory effect of Y0-C10-HSL on the formation of extracellular polysaccharides via QS, the structure of the biofilm is compromised, thus affecting biofilm formation and dispersion. Exopolysaccharides are largely involved in the surface attachment, formation, and stabilization of biofilm structure. Therefore, the importance of exopolysaccharides is self-evident. In this study, we found that Y0-C10-HSL also significantly inhibited the formation of total exopolysaccharides. Pili and flagella also function in biofilm formation during colonization and infection caused by *P. aeruginosa*. Type IV pili (T4P) can achieve strong adhesion between bacteria and host or abiotic surface through twitching motility. This relies on the extension–tethering–retraction–extension of T4P when responding to a chemical gradient [43,44]. Flagella push bacteria forward through rotation, mediate the swimming movement of *P. aeruginosa*, and provide mechanical and physical support during biofilm formation [45]. Both swimming and twitching motility of *P. aeruginosa* were considerably inhibited by Y0-C10-HSL. In the KEGG enrichment analysis of DEGs, genes related to pili and flagella were also counted, and they were found to be down-regulated.

**Figure 9:**
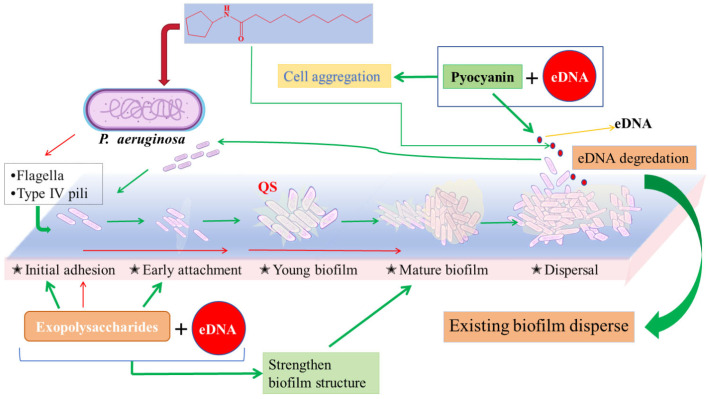
Mechanism of Y0-C10-HSL on *P. aeruginosa* biofilm. Red arrows indicate reverse regulation or inhibition, and green arrows indicate promotion or positive regulation.

Previous studies demonstrated that *phzABCDEFG* (pyocyanin synthesis genes) responded to the Las and Rhl systems and that their maximal expression required both systems [39,46,47]. In our study, *phzA*, a gene related to pyocyanin synthesis, the main pathogenic factor of *P. aeruginosa*, was also found in the KEGG of down-regulated DEGs. Pyocyanin is capable of binding to eDNA and causing the solution to become more viscous, thereby strengthening the physical–chemical relationships between the biofilm matrices and the surrounding environment, as well as leading to cell clustering [8]. As mentioned above, if eDNA is degraded and the formation of pyocyanin is inhibited, it jointly affects the aggregation of bacterial cells and further affects the biofilm formation (Figure 9). Pyocyanin production mainly receives regulation by the PQS system [9,48]. In the *P. aeruginosa* QS systems, Las is at the top of the QS hierarchy and is required for the optimal activation of the PQS system [2,49]. We speculated that Y0-C10-HSL may first affect the Las system, which in turn has an influence on the PQS system, inhibiting its virulence traits. This conjecture is similar to the findings of Francesca D’Angelo et al. [9]. Through the analysis of transcriptome results, it was found that Y0-C10-HSL inhibited the expression of the QS system gene in *P. aeruginosa*, thus affecting the biofilm. In other words, Y0-C10-HSL has two effects on biofilm, which not only inhibits the formation, but also has a good dispersion effect, indicating that it is a low probability event that *P. aeruginosa* produces adaptive resistance to it. *P. aeruginosa* biofilm can acquire drug resistance and overcome host immune defense [50]. The model experiment of *C. elegans* infection also proved that Y0-C10-HSL had a good antibacterial effect. This may be a result of the synergistic mechanism of Y0-C10-HSL on *P. aeruginosa* biofilm formation and dispersal, resulting in high nematode survival rates.

Amide bonds are widespread in nature, and due to their unique biochemical properties, they are important indicators for screening bioactive molecules in many marketed drugs and clinical candidates [51]. A distinctive feature of pharmaceuticals in therapeutic areas such as antibacterial, antioxidant, anti-diabetic, and anti-cancer treatment is that the vast majority of them have an amide structure. In this study, we targeted the quorum sensing system and synthesized Y0-C10-HSL (N-cyclopentyl-n-decanamide) as a potent QSI of *P. aeruginosa*, which could provide a novel foundation for coping with *P. aeruginosa* infections.

## 4. Materials and Methods

### 4.1. Chemical Reagent

Reactants and solvents used were the following: cyclopentylamine, decanoyl chloride, dichloromethane, ethylacetate, petroleum ether, dimethyl sulfoxide (DMSO), and dimethyl sulfoxide-D6. They were purchased from Shanghai MΛCLIN Biochemistry Co. (Shanghai, China) Solvents and chemical reagents were purchased and used without purification.

### 4.2. Bacteria and Cultivation

*P. aeruginosa* PAO1 (ATCC15692) was purchased from American Type Culture Collection (ATCC) and *Escherichia coli* (*E. coli*) OP50 was purchased from China General Microbiological Culture Collection Center (Beijing, China). *P. aeruginosa* and *E. coli* were incubated in Luria–Bertani (LB) liquid or solid medium (yeast extract 5 g/L, tryptone 10 g/L, NaCl 10 g/L, pH 7.0), and the incubator was set at 180 rpm and 37 °C. *Caenorhabditis elegans* (*C. elegans*) was cultivated at a constant temperature of 20 °C on Nematode Growth Medium (NGM) plates coated with *E. coli* OP50 as the standard food source.

### 4.3. Design and Synthesis of Y0-C10-HSL

**Figure 10:**
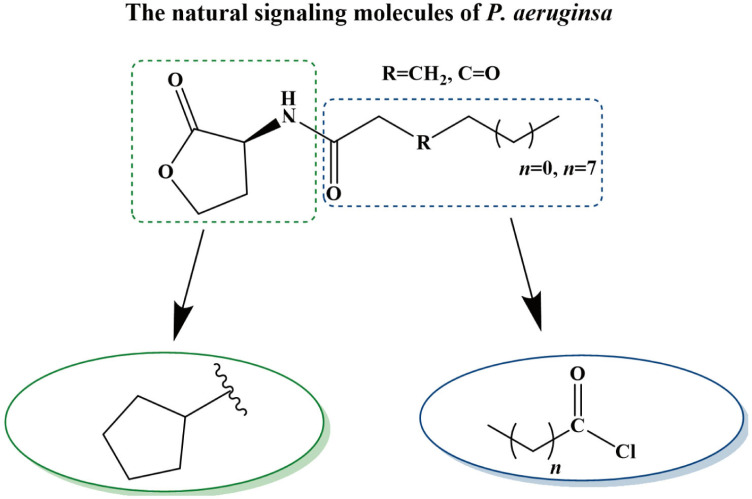
Design ideas of quorum sensing analogues.

The design idea of N-cyclopentyl-n-decanamide (Y0-C10-HSL) is shown in Figure 10. Y0-C10-HSL was synthesized by a chemical method to change the homoserine lactone ring and acyl side chain based on the natural signaling molecules (3-O-C_12_-HSL and C_4_-HSL) at the same time.

The compound was synthesized as reported previously with some modifications [39,52]. Cyclopentylamine (8.5 g, 100 mmol) was added to a round-bottomed flask containing 10 mL CH_2_Cl_2_ under ice bath conditions, and then decanoyl chloride (1.95 g, 10 mmol) was added drop by drop. After a 30 min reaction period, the ice bath was removed, and the reaction continued for 14 h with stirring at room temperature. When the reaction was completed, the organic phase was sequentially washed with 5% sodium bicarbonate solution, 1 mol/L hydrochloric acid, and a saturated NaCl solution (in a 1:1 volume ratio), each wash being repeated three times. Throughout this washing process, the agent was separated and purified by extraction. Anhydrous MgSO_4_ is added to the organic phase, which contains the target compound, with the objective of removing residual water. Following this, the organic phase is concentrated under reduced pressure, with the aim of obtaining the target product. To achieve further purification of the target product, a silica chromatography column and the following eluent systems were employed. The eluent system comprised the following proportions (*v*/*v*): 1:5, 1:4, 1:3, 2:3, 1:1, 3:2, and 1:0 ethyl acetate/petroleum ether. The eluate containing the target product was collected and concentrated again under reduced pressure, thereby obtaining the pure target product. Reactions were monitored with thin-layer chromatography. The final products were subjected to analysis by ^1^H NMR on a superconducting nuclear magnetic resonance spectrometer (BRUKER 500 MHz AVANCE NEO, Bremen, Germany) and subsequently characterized structurally using MestReNova (Version 14.0). The melting point was determined by the X-4 digital-display micro-melting point tester (Beijing Tektronix Instrument Co., Ltd., Beijing, China). The agent was stored at −20 °C.

N-cyclopentyl-n-decanamide: yield 87%; white powder, melting point 44~48 °C; purity > 95%; ^1^H NMR (500 MHz, chloroform-d) δ 5.35 (s, 1H), 4.21 (q, J = 7.0 Hz, 1H), 1.99 (dq, J = 12.5, 6.3 Hz, 2H), 1.85–1.46 (m, 8H), 1.37–1.14 (m, 14H), 0.88 (t, J = 6.8 Hz, 3H); ^13^C NMR (126 MHz, chloroform-d) δ 172.69, 76.77, 51.05, 37.03, 33.21, 31.87, 29.46, 29.37, 29.30, 29.27, 25.86, 23.72, 22.67, 14.11. The hydrogen spectrogram and carbon spectrogram exhibited a profile that was consistent with that of the desired compound and was designated Y0-C10-HSL (Figures S1 and S2).

### 4.4. Growth Curves

*P. aeruginosa* was cultured to the logarithmic growth stage. Subsequently, 5 mL was aspirated and placed into 45 mL fresh PPGAS medium (MgSO_4_·7H_2_O 0.396 g/L, Tris 14.54 g/L, KCl 1.19 g/L, glucose 5 g/L, NH_4_Cl 1.07 g/L, peptone 10 g/L, Tris 14.54 g/L, pH 7.2). Then, Y0-C10-HSL was added (0, 10, 100, and 200 μmol/L, respectively). Finally, the suspension was incubated for 24 h at 37 °C and 180 rpm. The optical density of the cultures was monitored at OD_600_ every 2 h for a total of three replicates [51,53].

### 4.5. The Effect on Biofilm

#### 4.5.1. Biofilm Formation

The biomass of the biofilm was assessed using the crystal violet staining method [54,55]. The bacterial suspension in the logarithmic growth phase was diluted with fresh PPGAS medium to an OD_600_ equal to 0.01. Subsequently, Y0-C10-HSL was added (0, 10, 100, and 200 μmol/L, respectively). Then, 160 μL of bacterial suspension was added into 96-well plates (Corning Incorporated, Kennebunk, ME, USA) and 6 parallel wells were made for each group. The plates were cultured under static condition at 37 °C for 24 h. Bacterial suspension was slowly removed and the biofilm was rinsed three times with sterile PBS buffer. Next, the biofilm was stained with 0.5% crystal violet for 15 min, and the excess crystal violet was removed with PBS buffer. The samples were dried at 25 °C, 33% acetic acid was added to dissolve the crystal violet, and OD_570_ was measured by a microplate reader.

#### 4.5.2. Dispersion of Pre-Formed Biofilm

The biofilm was assessed with the similar approach mentioned above. Six parallels were set up for each group. An amount of 160 μL of bacterial suspension (OD_600_ = 0.01) was transferred to a 96-well cell culture plate and incubated at 37 °C. After incubating for 24 h, the upper layer was carefully aspirated and the biofilm was rinsed. Subsequently, 160 μL of PPGAS medium containing 0, 10, 100, and 200 μmol/L Y0-C10-HSL, respectively, was added to the wells. Next, the 96-well cell culture plate was cultured under static condition at 37 °C for 24 h. Finally, the medium was removed and the biofilm was gently rinsed three times. The subsequent procedure is identical to that described in Section 4.5.1.

#### 4.5.3. Biofilm Structure

The coverslips were autoclaved and placed in the 6-well plate. Then, 2000 μL of bacterial suspension (1 × 10^7^ CFU/mL, containing 0, 10, 100, and 200 μmol/L Y0-C10-HSL, respectively) was added to each well. Each group was set three parallels. After static incubation at 37 °C for three days, the medium was removed and the biofilm was rinsed. The rinsed biofilm was fixed with 2.5% glutaraldehyde overnight. Subsequently, the biofilm was washed and dehydrated with gradient ethanol (50%, 70%, 80%, 90%, 95%, 100%) for 15 min [56,57]. Finally, the biofilm was dried with a vacuum freeze dryer (Xinzhi, Scientz-30YG/A, Ningbo, China), and the morphology and structure of the biofilm was observed with SEM (ZEISS GeminiSEM 500, Carl Zeiss, Oberkochen, Germany), where the magnification was 4000× [58].

### 4.6. Exopolysaccharides Assay

The drug concentration, bacterial density, and culture conditions were as previously described in Section 4.5.1, with the exception that the culture system was 50 mL in volume. The extraction of extracellular polymeric substances (EPSs) was conducted in accordance with the methods previously outlined by Shen et al. and Zhou et al. [59,60]. First, the bacterial suspension was centrifuged at 15,000× *g* for 15 min to collect the bacterial precipitate. Secondly, an equivalent volume of sterile water was added and heated at 80 °C for 30 min, and then the supernatant, EPS, was centrifuged and saved. The quantification of exopolysaccharides was evaluated by the phenol–sulfuric acid (PSA) method [61,62]. Specifically, 1000 μL supernatant was mixed with 500 μL 6% phenol and 2500 μL 95% sulfuric acid. After shaking and cooling, OD_490_ was measured. The EPS extract supernatant was frozen at −80 °C for 10 min and was lyophilized for 24 h by a vacuum freeze dryer. The dried EPS samples were then fully ground with potassium bromide in a 1:100 (m/m) ratio until the particle size was less than 2 μm. The chemical groups on the surface of the samples were detected by an FT-IR (VERTEX 70, Bruker, Bremen, Germany) spectrometer in the range of 4000–400 cm^−1^ after pressing [63].

### 4.7. The Motility Assay

#### 4.7.1. Swimming Motility

The autoclaved swimming medium (LB agar medium containing 0.3% agar) was cooled to about 55 °C and Y0-C10-HSL was added to the final concentrations of 0, 10, 100, and 200 μmol/L, respectively. After thorough mixing, the medium was used to prepare solid plates. Sterile toothpicks were used to inoculate 2 μL *P. aeruginosa* suspension into the middle layer of the medium [64]. The effect of Y0-C10-HSL on swimming motility was assessed by measuring the diameter of circular turbidity circles of the colonies in the agar layer after incubating for 24 h.

#### 4.7.2. Twitching Motility

Twitching motility medium (LB agar medium containing 1.2% agar) was made as described in Section 4.7.1. The plates were allowed to solidify and inverted overnight, and the next day sterile toothpicks were used to inoculate a suspension of *P. aeruginosa* into the medium (punctured to the bottom). After incubating for 48 h, the agar was gently scraped off with a scraper and tweezers, and then the twitching motile zone on the plate was stained with 0.5% crystal violet for 30 min and finally washed to remove free crystal violet. The distance traveled from the point of inoculation to the edge of movement was recorded to evaluate twitching movement [64,65].

### 4.8. C. elegans Survival Assay

The establishment of the *C. elegans* infection model was based on the previously described method with slight modifications [66,67]. To begin with, NGM plates containing 0, 10, 100, and 200 μmol/L Y0-C10-HSL, respectively, were manufactured. They were inoculated with *E. coli* OP50 or *P. aeruginosa* and labeled in groups. The inoculated NGM plates were incubated for 24 h. Finally, 100 synchronized L4-stage N2 nematodes were picked onto NGM plates, sealed, and incubated in a biochemical incubator (Bruepard Instruments, Shanghai Yiheng Instrument Science Co., Shanghai, China) at 20 °C for 80 h. Observations were recorded every 8 h during the incubation period.

### 4.9. Transcriptomic Analysis

#### 4.9.1. RNA Extraction and cDNA Library Construction and Sequencing

*P. aeruginosa* was cultivated in liquid medium with Y0-C10-HSL concentrations of 0 and 200 μmol/L. After 24 h of cultivation, an appropriate volume of bacterial suspension was aspirated and centrifuged at 15,000× *g* for 15 min at 4 °C. Discard the supernatant and retain the bacterial precipitate. An amount of 75% ethanol was added to the bacterial precipitate and then placed at −80 °C overnight for the purpose of inactivation. The next day, the ethanol was removed from the samples and the samples were quickly snap-frozen in liquid nitrogen and then placed at −80 °C. RNA was extracted with TRIzol reagent (Invitrogen, Carlsbad, CA, USA) and further purified using RNase-free DNase I (Epicentre, Madison, WI, USA) and Ribo-Zero™ rRNA Removal Kit (Epicentre) to obtain the ideal RNA sample. The cDNA library was constructed and sequenced using strand-specific RNA-Seq by Majorbio (Shanghai, China)

#### 4.9.2. Gene Expression Analysis

The quantification metric used was TPM (transcripts per million reads). Gene expression was quantified with the help of RSEM (http://deweylab.github.io/RSEM/ (accessed on 24 February 2024)). Differential gene expression between samples was analyzed utilizing the DESeq2 package, where statistical significance was set at a *p*-value < 0.05 and a fold change (FC) > 1.5 for the identification of significantly differentially expressed genes (DEGs). We then performed Gene Ontology (GO) and Kyoto Encyclopedia of Genes and Genomes (KEGG) enrichment analyses on the DEGs identified at this threshold with the aim of elucidating the biological functions and pathways primarily affected by these differentially expressed genes.

### 4.10. Statistical Analysis

All dates are presented as mean ± standard deviation (mean ± SD), and each experiment was performed in triplicate. GraphPad Prism 8 v8.0.2 was utilized to analyze and plot the experimental results. Statistical differences were calculated via *t*-tests and *p* < 0.05 is deemed to be statistically significant. Nematode survivals were analyzed using the log-rank test.

## Figures and Tables

**Table 1:** Chemical structure of quorum sensing signal molecule in *P. aeruginosa* PAO1.

System Category	Chemical Structure	Signal Molecule Name
Las	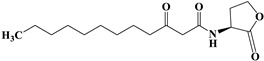	N-3-oxo-dodecanoyl-L-hydroserine lactone (3-O-C12-HSL)
Rhl	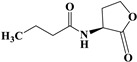	N-butyryl-L-hydroserine lactone (C4-HSL)
PQS	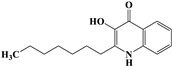	2-heptanyl-3-hydroxy-4-quinolone (PQS)
IQS	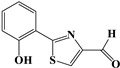	2-(2-hydroxyphenyl)-thiazol-4-aldehyde (IQS)

**Table 2:** Differentially expressed genes related to quorum sensing system.

Description	Gene Name	Log2FC	Function Description
Quorum sensing	*BGV84_RS03095*	−1.143050622	ABC transporter permease
	*BGV84_RS03090*	−1.078897352	ABC transporter substrate-binding protein
	*BGV84_RS25465*	−0.617002839	branched-chain amino acid ABC transporter substrate-binding protein
	*BGV84_RS19945*	−0.774877297	branched-chain amino acid ABC transporter substrate-binding protein
	*pmrB*	−0.758269466	two-component system sensor histidine kinase PmrB
	*livG*	−0.651935974	high-affinity branched-chain amino acid ABC transporter ATP-binding protein LivG
	*BGV84_RS10805*	−1.214829414	3-deoxy-7-phosphoheptulonate synthase class II
	*phzA*	−1.214829414	phenazine biosynthesis protein PhzA
	*fadD1*	0.952214648	long-chain-fatty-acid–CoA ligase FadD1
	*BGV84_RS03100*	−0.742557348	ABC transporter permease
	*crp*	−0.642262549	cAMP-activated global transcriptional regulator CRP
	*BGV84_RS17185*	−0.713945732	AMP-binding protein
	*potA*	−0.190985405	polyamine ABC transporter ATP-binding protein
Flagellar assembly	*fleP*	−0.604913812	pili length/flagellar attachment protein FleP
	*BGV84_RS01600*	−0.652319441	cystine ABC transporter substrate-binding protein
	*BGV84_RS23030*	−0.697947637	RNA polymerase factor sigma-54
Biofilm formation	*BGV84_RS17725*	−0.61142072	Hcp family type VI secretion system effector
	*BGV84_RS13310*	−0.761199559	Hcp family type VI secretion system effector
	*tssC*	−0.734929743	Hcp family type VI secretion system effector
	*crp*	−0.642262549	cAMP-activated global transcriptional regulator CRP

## Data Availability

All the sequencing data involved in this paper have been uploaded to the SRA database under accession number PRJNA1082122 (https://www.ncbi.nlm.nih.gov/bioproject/PRJNA1082122 (accessed on 24 February 2024)).

## Supplementary Materials

The following supporting information can be downloaded at https://www.mdpi.com/article/10.3390/ph17121719/s1. Figure S1. Y0-C10-HSL ^1^H NMR (500MHz, Chloroform-d). Figure S2. Y0-C10-HSL ^13^C NMR. Figure S3. Correlation plot of gene expression between samples. Table S1. The quality of total RNA samples. Table S2. Quality control of sequencing data from sample. Table S3. The mapping rate of samples and reference genomes. Table S4. Differentially expressed genes.
