# Supplementary material for: Transcriptome Analysis Reveals the Mechanism of Y0-C10-HSL on Biofilm Formation and Motility of Pseudomonas aeruginosa
Source: Pharmaceuticals (Basel). 2024 Dec 19;17(12):1719. doi: 10.3390/ph17121719 (PMC11678461; doi:10.3390/ph17121719)
Supplement: Supplementary file 1 [file pharmaceuticals-17-01719-s001.zip › pharmaceuticals-3200616-supplementary.pdf]

Transcriptome Analysis Reveals the Mechanism of Y0-C10-HSL on Biofilm Formation and Motility of *Pseudomonas aeruginosa*

Deping Tang [1], Yali Liu [1], Huihui Yao [1], Yanyan Lin [1], Yanpeng Xi [1], Mengjiao Li [1] and Aihong Mao [1,2,*]

1 School of Biological & Pharmaceutical Engineering, Lanzhou Jiaotong University, Lanzhou 730070, China; tangdp@mail.lzjtu.cn (D.T.)

2 Gansu Provincial Academic Institute for Medical Research, Lanzhou 730050, China

* Correspondence: maoaih@aliyun.com

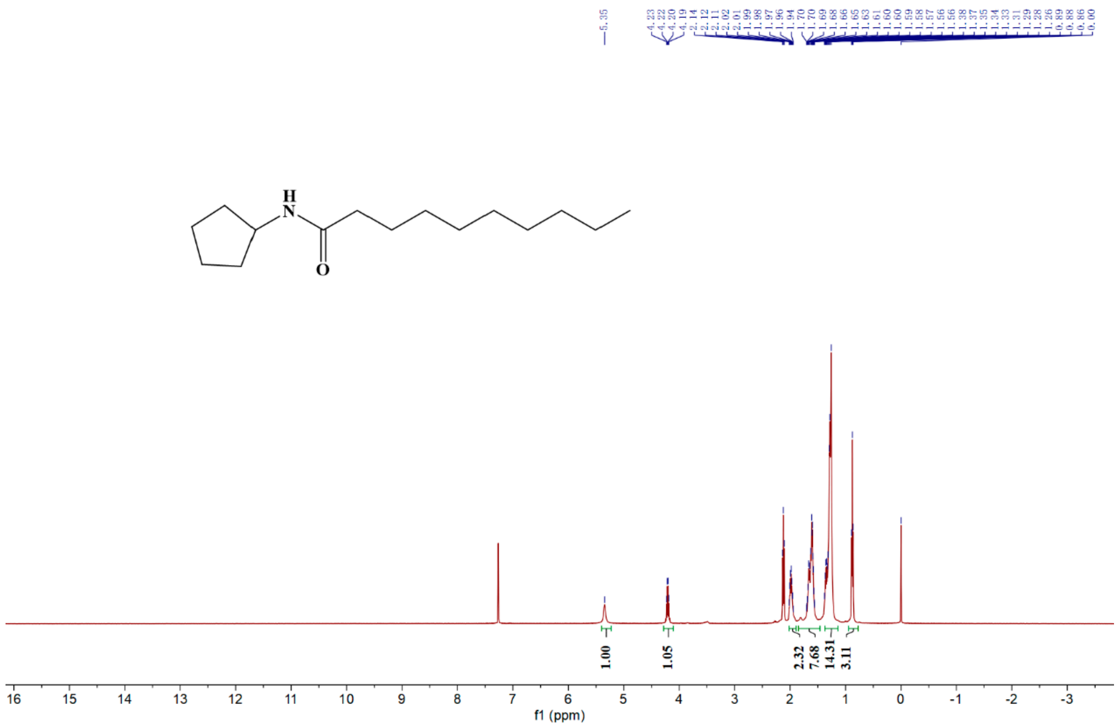

Figure S1. Y0-C10-HSL ^{1}H NMR (500MHz, Chloroform-d).

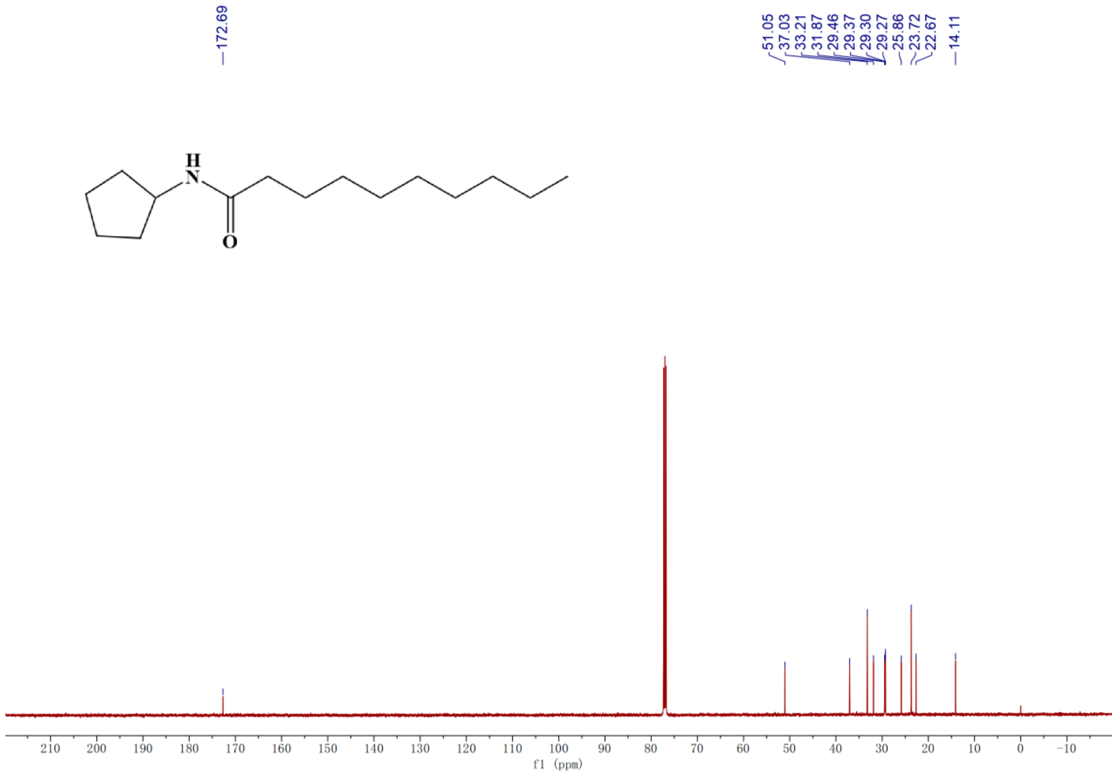

Figure S2. Y0-C10-HSL ^{13}C NMR (126 MHz, Chloroform-d).

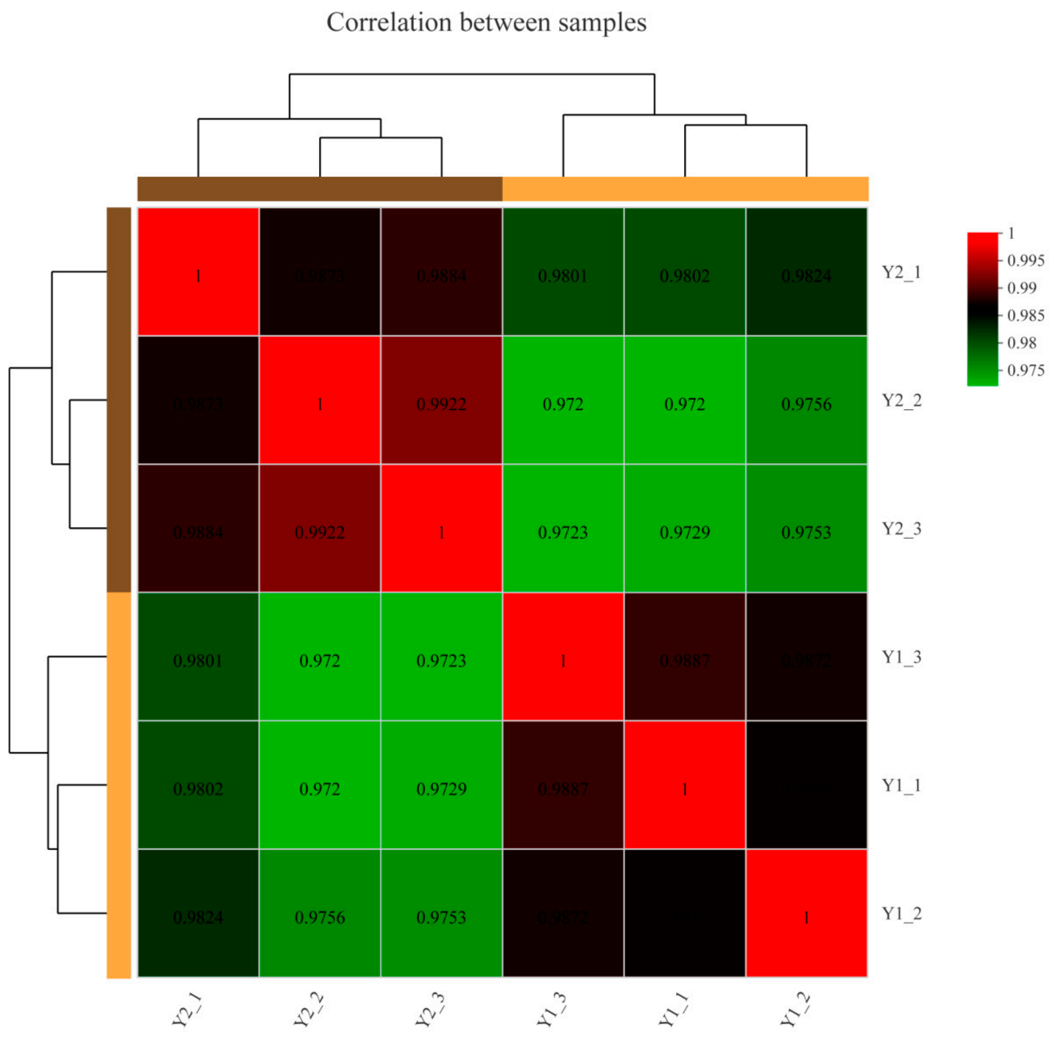

Figure S3. Correlation plot of gene expression between samples.

Table S1. The quality of total RNA samples.

Sample name	Concentration (ng/μL)	Total (μg)	OD260/OD280	OD260/230	RIN
Y1_1	210.60	7.37	2.09	2.21	9.80
Y1_2	327.60	11.47	2.07	2.24	9.80
Y1_3	340.90	11.93	2.06	2.22	9.80
Y2_1	507.00	17.75	2.14	2.35	9.40
Y2_2	498.40	17.44	2.12	2.21	9.60
Y2_3	161.50	5.65	2.04	2.06	9.60

Table S2. Quality control of sequencing data from sample.

Sample Name	Raw Bases (bp)	Raw Q20 (%)	Raw Q30 (%)	Clean Bases (bp)	Clean Q20 (%)	Clean Q30 (%)
Y1_1	4717467406	96.65	92.81	4046527151	97.83	94.65
Y1_2	4623755296	96.88	93.04	3860488405	98.02	94.92
Y1_3	4409595318	95.67	91.5	3504419525	97.9	94.67
Y2_1	4261108562	96.56	92.64	3589735659	97.91	94.72
Y2_2	4598804660	96.74	92.86	3940962768	97.84	94.6
Y2_3	3831863278	96.67	92.8	3303911218	97.86	94.59

Table S3. The mapping rate of samples and reference genomes.

Sample Name	Total Reads	Genome Mapped Ration (%)	Unmapped Reads Ration (%)	Uniq Mapped Ration (%)
Y1_1	30575976	85.05	14.95	82.38
Y1_2	30234154	84.89	15.11	82.15
Y1_3	28727786	85.55	14.45	82.43
Y2_1	27882926	83.95	16.05	80.94
Y2_2	30104632	84.24	15.76	81.23
Y2_3	25103302	83.85	16.15	81.14

Table S4. Differentially expressed genes

Differential expressed genes	Genes
Expressed mRNA and sRNA	585
Up-regulated mRNA and sRNA	254
Down-regulated mRNA and sRNA	331
Expressed mRNA	549
Up-mRNA	245
Down-mRNA	304
Expressed sRNA	36
Up-regulated sRNA	9
Down-regulated sRNA	27
